# Walk, Look, Remember: The Influence of the Gallery’s Spatial Layout on Human Memory for an Art Exhibition

**DOI:** 10.3390/bs4030181

**Published:** 2014-07-08

**Authors:** Jakub Krukar

**Affiliations:** Department of Architecture and the Built Environment, Northumbria University, NE1 8ST Newcastle-upon-Tyne, UK; E-Mail: jakub.krukar@northumbria.ac.uk

**Keywords:** memory, visual attention, spatial cognition, spatial layout, space syntax, isovist, museum

## Abstract

The spatial organisation of museums and its influence on the visitor experience has been the subject of numerous studies. Previous research, despite reporting some actual behavioural correlates, rarely had the possibility to investigate the cognitive processes of the art viewers. In the museum context, where spatial layout is one of the most powerful curatorial tools available, attention and memory can be measured as a means of establishing whether or not the gallery fulfils its function as a space for contemplating art. In this exploratory experiment, 32 participants split into two groups explored an experimental, non-public exhibition and completed two unanticipated memory tests afterwards. The results show that some spatial characteristics of an exhibition can inhibit the recall of pictures and shift the focus to perceptual salience of the artworks.

## 1. Introduction

It is difficult to establish what constitutes a good museum/gallery exhibition. Yet, it has been widely acknowledged that what visitors attend to [1,2] and what they “get out of it” [3] (p. 46) should be a priority. To facilitate this, the curator’s main task lies in presenting the exhibited material within the space available. By doing so, the curator must inevitably influence the perceived importance and possible interpretations of the object, which often go beyond the original intentions of the artist [4]. Being aware of the space’s characteristics is therefore crucial when designing an exhibition with its visitors in mind [5]. Consequently, providing exhibition designers and curators with tools to understand and control the influence of the already existing layouts upon the final experience of their visitors is an important goal, potentially serving in the design of new galleries in the future.

The relevant cognitive processes are *visual attention* and *memory*, and their role has been widely studied in the fields of visitor studies and experimental aesthetics [1,2,6,7,8]. Visual attention is often held to be a bottom-up directed process, driven by the external features of the environment, such as the objects’ visual salience [9]. However, growing evidence shows our visual perception is guided by higher cognitive processes to regions which store information important for our current goal—a top-down strategy [10]. The primary purpose of an art gallery is to view objects within a space. This makes them an ideal research setting for investigating the influence of space on human cognition; in other building types, those “cognitive aims” are rarely so uniform. Also, visiting museums has remained a popular activity despite the widespread availability of reproductions online. One significant reason for this might be that a dynamic spatial experience is superior to memorising a list of the same objects [11]. There is no doubt that museum spaces are not experienced as simply neutral white cubes (see [12] for the discussion on the subject from the artist’s viewpoint). Disentangling this cognitive experience, with the emphasis on its spatial predictors, is the main challenge of this work.

The mechanisms of spatial cognition that guide our exploration of space and spontaneous memory of encountered objects have been largely investigated in the landmark literature. If we define landmarks as easily recognisable objects serving as a point of reference in space [13] and we assume that their acquisition is spontaneous [13,14], then the findings from these studies could be applied to artworks in a gallery space. Even during a free exploration of an art gallery, visitors must still use their spatial abilities to orientate themselves in space. If this was not true, their movement would be random, with no mechanisms helping them to avoid revisiting rooms, to explore new spaces, and to find one’s way out. Viewing art in a museum must be inextricably bounded with acquiring knowledge about spatial location of certain objects and the gallery’s layout. For this reason, it is important that studies of landmarks make the distinction between *object-based* and *location-based* attention [15], resulting in the *knowledge of objects* and *knowledge of the spatial relations between them* [16].

For instance, Janzen [17] observed that objects placed next to decision points (junctions) in a virtual museum are recalled faster on a computer-based recognition task than those placed along straight paths. The author suggested that this effect might be the result of a linkage between the memory representation of a particular object and the representation of its location. This explanation would be in line with different neural activity patterns in the parahippocampal gyrus (responsible for place-object mapping), that can be induced by decision-point-based and non-decision-point-based objects [14]. In a later study, Miller and Carlson [18] designed a similar virtual museum to Janzen’s, but in addition aimed to take the objects’ perceived salience into account. Such salience, in the real-life context, is likely to be dependent on many more factors than just visual dominance (e.g., emotional reaction, or previous knowledge/memories about the object). For this reason, objects placed inside were separately rated for their perceived salience by an independent group of participants. The authors managed to replicate Janzen’s [17] results when objects of high perceived salience were placed on decision points, but did not when highly salient objects were purposefully placed on navigationally irrelevant locations (*i.e.*, on non-decision points). In the latter variation of the experiment, high perceived salience was a factor enhancing response times on the computer-based recognition test, while navigational relevance guided participants’ responses in map drawing and route description tasks. As the authors conclude, the encoding of a landmark (object) might be driven by its perceptual features, whereas its selection during spatial tasks seems to be driven by its spatial features [18]. This shows the importance of separating the *object-oriented* and *location-oriented* memory in this context. Miller and Carlson’s (ibid.) results also suggest that the former should be highly dependent on the objects’ perceived salience, while the latter should remain unrelated to it.

While the qualitative distinction between those memory types seems fully applicable to the art gallery experience, the specific factors having an effect on the strength of those memories is likely to differ. Navigational behaviour highly varies depending on the actual context, as well as goals and strategies of the individuals [19]. A gallery visitor is likely to direct his/her attention to artworks, and in a visually ascetic space, these become highly salient reference points, but their navigational importance is secondary. Instead, the spatial relations between them can become an important component of the viewer’s understanding of curatorial intentions (either explicitly or spontaneously). A gallery visit therefore incorporates both types of attention: *object-based* and *location-based*. These induce memory traces of the individual objects, as well as of the spatial relations between them. Object-based memories should therefore mainly (but not exclusively) be influenced by the picture’s perceived salience. Location-based memories are dependent on the pictures’ position in the gallery, and therefore derive from initially designed curatorial narrative [20]. If the effect of spatial location in landmark studies depended on its importance for navigational decisions, perhaps in the art-viewing context this effect would be influenced by the location’s importance for potential understanding of the narrative of the exhibitions (such as co-visibility of multiple artworks with it).

If this provides a partial understanding of the visitor’s experience, the exact influence of space remains unclear. Curators have developed many strategies for displaying art, but rarely had the opportunity to verify their effects empirically [7]. The architectural field of Space Syntax has investigated different approaches to the use of space for curatorial narrative. It has been analysed both at the level of global properties of the whole galleries, as well as the local spatial characteristics of specific artwork locations. Space Syntax studies showed, for instance, how the way knowledge is transmitted can be reflected in the spatial logic of the exhibits [21], or how visitor movement patterns are affected by the spatial layout [22]. Many case studies highlighted categorical segregation of artefacts in space and syntactic intelligibility of museum buildings (e.g., [20,23,24,25]; see [26] for an overview). By doing so, they showed that museum buildings significantly differ on the spectrum of their spatial organisation.

The focus of this paper is mainly on the local properties of artworks’ locations which determine their spatial and, as a consequence, curatorial relationships with each other. These spatial properties have been shown to differ significantly across museums. Tzortzi [27], for example, showed how the placement of pictures and the overlap of their visual fields can be used in the creation of the final gallery experience. These spatial practices can also distinguish between those museums imposing some pre-defined meaning upon the visitor’s interpretation, and those which allow for more unrestricted explorations [28]. Further, Stavroulaki and Peponis [29] suggested that the positioning angle of artworks is an important factor influencing paths taken and, as a result, the final experience of the visitors. Since looking at sculptures and paintings is the main goal of a museum visit, most visitors will attempt to position themselves within a comfortable viewing position in front of these works. A visual field restricted to a 60-degree visibility cone in front of an artwork is suggested as such a “catchment” area [29]. Therefore, the spatial position of artefacts in a museum must have a behavioural effect upon visitors, thus resulting in a different experience on the cognitive level. A link towards the empirical confirmation of these suggestions has already been made by Wineman *et al.* [30], who measured the sequence of visitors’ engagement with the same science exhibits in differently organised spatial environments. They emphasise that the cognitive effect of spatial exhibition design goes far beyond the curatorial narrative and can be an independent medium for construction of meaning.

As it has been shown, landmark studies distinguished between two types of memory playing a significant role in the visitor experience in a virtual museum space: the memory of objects, and memory of their spatial location. However, the analysed spatial properties of those objects were limited to decision and non-decision points in the layout. Space Syntax studies explored more complex spatial properties of museum exhibitions, taking into account both global relations between various parts of the building, as well as local properties of single exhibits, such as their visibility. Those findings, however, came mostly from case studies, and cannot be widely generalised. Three other papers addressed this issue using the experimental method, seeking the causal relationship between more complex spatial properties and their influence on human cognition.

Wiener *et al.* [31] showed how visibility properties are related to participants’ navigational behaviour and environmental ratings, proving the linkage between architectural shape and human cognition. Another experiment in Human–Computer Interaction [32] showed the influence of various placement properties on the memory of passersby for the content of public screen displays. The most recent contribution signified the importance of co-visibility of many pictures from various positions in space on categorical judgments made by the gallery visitors [33].

The purpose of this study is to establish a causal relation between the local spatial properties of art exhibits and the cognitive processes which contribute to the museum experience. This is achieved by joining experimental design with Space Syntax analyses, and measuring the cognitive outcomes of a gallery visit.

## 2. Method

A study was designed in which participants explored an especially designed art gallery and took part in two unanticipated memory tests. Free exploration was suggested, in contrast to many experimental designs that restrict the participants by imposing a pre-defined path or walking pace, or by showing a video clip of the route instead.

### 2.1. Space and Materials

**Figure 1:**
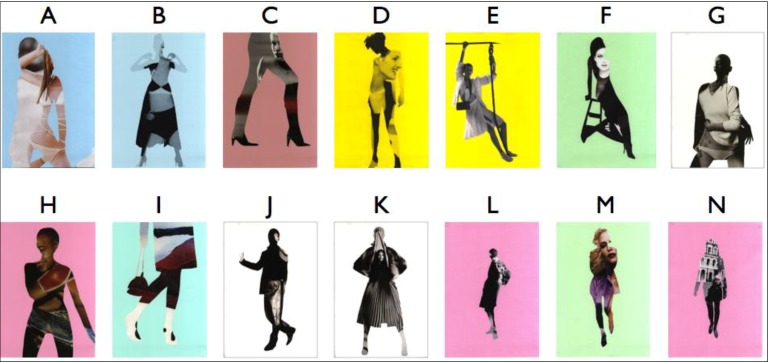
Pictures used in the study.

**Figure 2:**
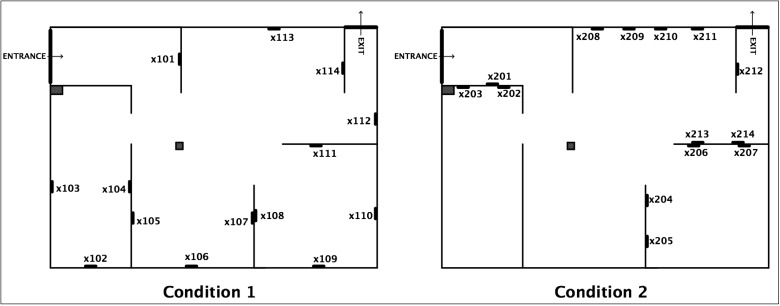
Spatial layout and location names in two experimental conditions.

Images used for the study were artworks of equal dimensions (portrait-oriented A3), created by local artist, Susi Bellamy (Figure 1) [34]. A non-public art gallery was arranged in a building otherwise used as a studio and exhibition space for fine arts students. Two experimental conditions were employed. The arrangement of the walls was identical in each condition, but the placement of pictures’ locations differed (Figure 2). Note that (as it will be shown quantitatively in Section 2.2) this is sufficient to create the diversity in visual measures, which are the main scope of this paper. After all, “it is this ordering of space that is the purpose of building, not the physical object itself. The physical object is the means to the end. (...) Buildings are not just objects, but transformations of space through objects.” [35] (p. 1). Therefore, the effect of this modification of spatial and visual relations (of the ordering of space) can be generalised to spatial layouts *per se*.

What people spontaneously memorise might result from the interaction of its spatial prominence and the stimulus’s perceived salience. For this reason, pictures’ perceived salience has been established in an independent study, and controlled (see Section 2.7). Moreover, in order to further separate the effect of space from the effect of pictures, the sequence of the pictures seen inside the gallery was randomised for each participant. Therefore, each visitor had seen the same pool of pictures (Figure 1) on the same set of locations (Figure 2), but in a unique, random combination. This allowed for recording the memory performance twofold: as a *picture-oriented* and *location-oriented* variable. It was hypothesised that significant correlation of location-oriented memory variables and the spatial properties of those locations would prevail, despite the influence of individual pictures’ perceived salience.

### 2.2. Space Syntax Measures

Spatial properties of each location were derived using Depthmap 10.14.00b [36]. The software was used for Visibility Graph Analysis (VGA), as well as to calculate the Boundary Visibility Graph (BVG) [37]. Visibility Graph is calculated by superimposing a grid on the analysed spatial layout. For each grid cell, the software “counts” how many grid cells can be visible from this point, given the walls’ restrictions. This results both in a mathematical measure of *Connectivity*, as well as its visualisation, where areas visible from the largest proportion of space are coloured red, and those which “see” the least (and concurrently can be “seen” from the smallest proportion of space) are coloured blue. This derives from an earlier concept of *isovist theory* [38], which is a polygon drawn on a building’s layout around a point of reference. This polygon covers the entire surface area visible from the given point. For the same point, its *isovist area* is therefore correlated with its VGA’s *Connectivity* value, the only differences being the resolution, the method of calculation, and units used. When derived from a reference point lying on the layout boundary, both of these measures essentially become an 180° isovist directed perpendicularly to the wall surface. The difference between VGA and BVG comes from the fact that BVG only considers grid cells lying along layout boundaries and therefore its *Connectivity* value is not equivalent to the location’s *isovist area**,*** but instead it reflects the proportion of other wall surfaces visible from the reference point. The potential importance of these measures comes from the fact that they describe which paintings are more likely to fall into the viewing field of a person randomly exploring the environment. As humans are sensitive to the isovist area and quite skilled at recognising the points in space which provide the largest isovist size [31], it could also be possible that artworks with larger isovist areas are associated with greater importance relative to the other parts of the exhibition.

Additionally, various isovist properties describing its size and shape were generated from each picture location—e.g., *area-to-perimeter* ratio and *point 2nd moment* [38] can describe how “spiky” the shape of a visibility field is. A “spiky” isovist would result in a less stable visual experience, whereby the connection between the viewer’s eyes and the object of reference is potentially often interrupted, e.g. by walls or columns. This property (under the combined concept of *jaggedness*) has already been linked with human spatial behaviour [31].

**Figure 3:**
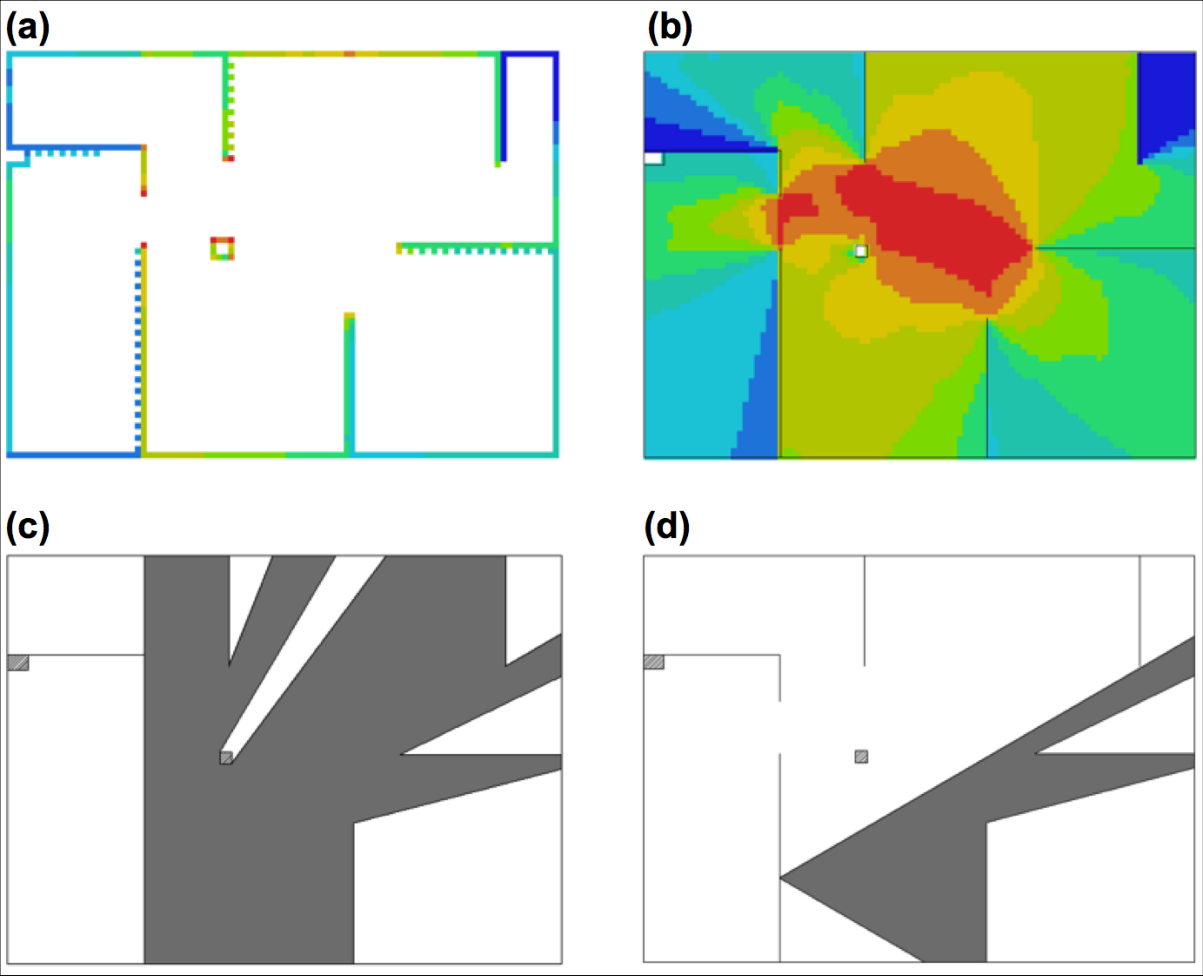
Some spatial analyses used in the study: **(a)** Boundary Visibility Graph (BVG); **(b)** Visibility Graph Analysis (VGA); **(c)** Sample isovist derived for location x105; **(d)** Sample Visibility Catchment Area (VCA), derived for location x105 (equal to its isovist restricted to a 60° cone).

For each picture location, two additional, non-standard variables were derived: the *number of other pictures present within the isovist* of each picture location and its *Visibility Catchment Area (VCA)*. The former indicates a potential co-visibility of other pictures with the analysed picture location (e.g., this number equals 2 for location x104 and 0 for x201). In the art gallery context, pictures’ co-visibility has been shown to influence viewers’ understanding of the exhibition [33]. The latter measure is equivalent to the area of an isovist generated from each location, but restricted by a cone of 60°. This partial isovist generated from the centre of the picture has been suggested for defining the area where viewers wish to position themselves in order to comfortably enjoy the visual experience provided by an artwork [29]. Note that such an area of comfortable viewing is a non-trivial issue and a subject of separate studies [39,40]. Figure 3 presents a visual example of few major analyses.

**Figure 4:**
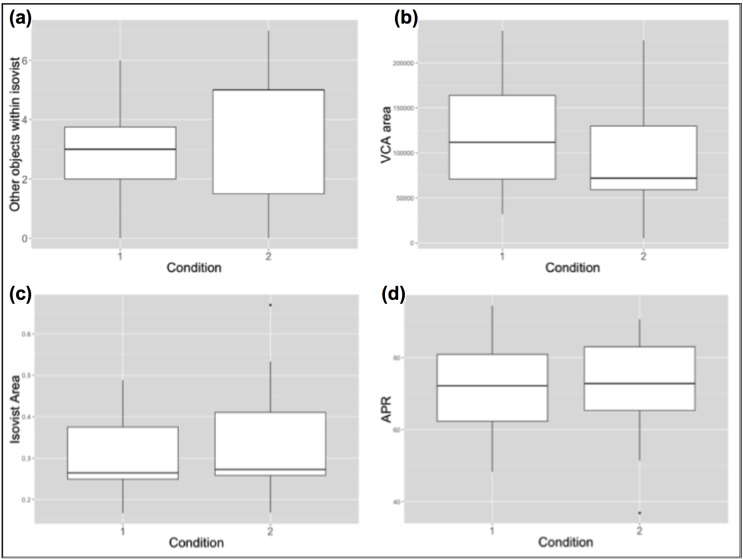
Boxplots presenting differences in spatial measures of picture locations between the two conditions: **(a)** Higher potential co-visibility in Cond; 2. **(b)** Lower average VCA in Cond; 2. **(c)** Similar isovist area sizes; **(d)** Similar area-to-perimeter ratios.

In order to quantify these spatial differences between Condition 1 and Condition 2 in aggregate, mean spatial properties of picture locations were compared across conditions (Figure 4). Firstly, mean VCA for locations in Condition 1 was larger (M = 117,464, SD = 63,268) than in Condition 2 (M = 99,261, SD = 69,807). Secondly, mean number of *other objects within single location’s isovist* was larger in Condition 2 (M = 3.86, SD = 2.35) compared to Condition 1 (M = 2.86, SD = 1.56), indicating that potential co-visibility of many pictures at the same time was higher in Cond. 2. On the contrary, the size and “spikiness” of isovist areas for locations in both conditions were similar. This confirms that both conditions allowed visitors to look at pictures from a similar proportion of the total gallery area. The difference was rather present in the quality of this visual experience—Condition 1, with higher *VCAs* and lower potential co-visibility should facilitate more comfortable and less distracted viewing.

### 2.3. Participants

Thirty-two participants, 13 female and 19 male, aged between 18 and 63 years (M = 30.75, SD = 11.73) with normal or corrected-to-normal vision were recruited through the university email system and local job-seeker internet discussion forums for a fee of £6. Before the experiment started, a short excerpt from a test for normal colour vision was administered to confirm the participant’s self-declaration [41]. Standard ethical procedures were employed throughout the study.

One participant declared previous familiarity with the gallery space and was removed from subsequent analyses, since wayfinding research had shown the influence of previous knowledge on spatial memory [42,43]. No artists or architects were allowed to participate.

### 2.4. Procedure

**Figure 5:**
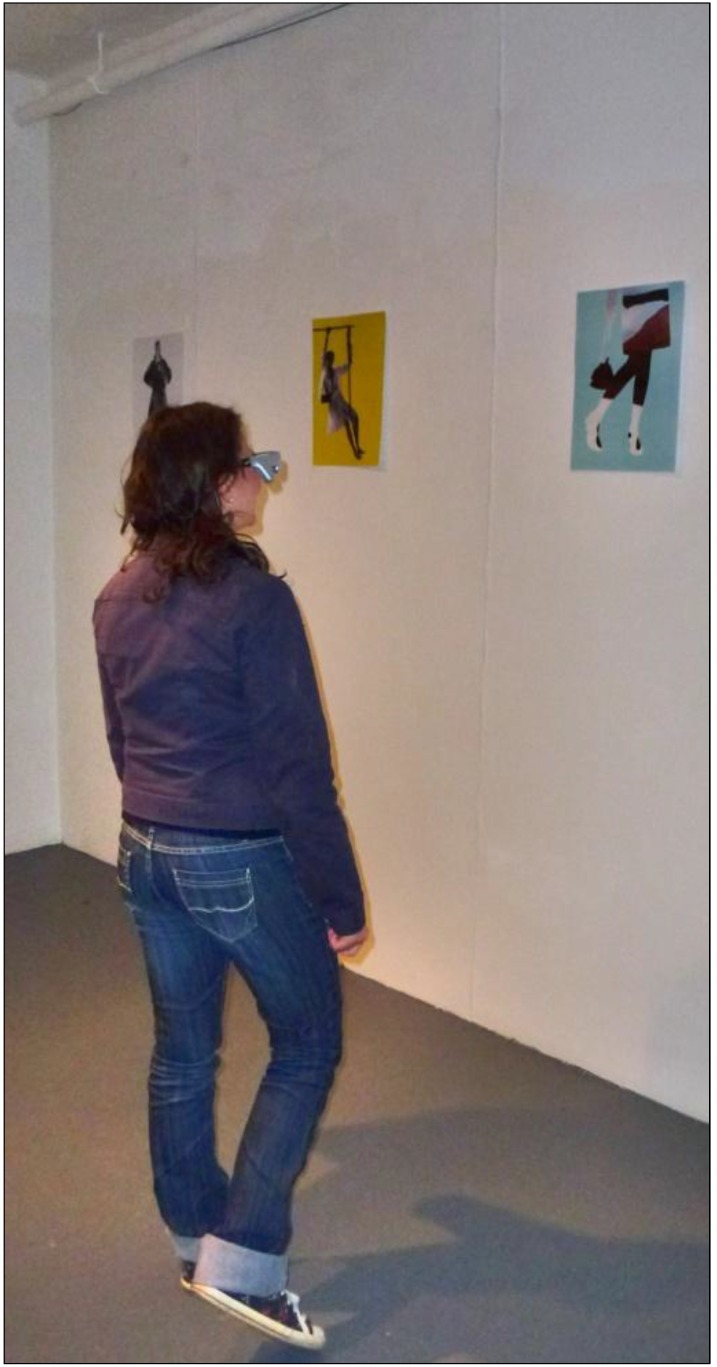
A simulation of the experimental procedure. Participants were wearing a mobile eye-tracking device, recordings of which are reported elsewhere [44].

Participants were randomly assigned to Condition 1 (N = 14), or Condition 2 (N = 17). They were asked to enter the main part of the gallery and to “explore it just as you would explore a regular art gallery” within the time limit of 30 minutes (Figure 5). The visitors were asked to look at each picture at least once, but there were no constraints regarding the trajectory taken, or revisiting the previously seen pictures. After exiting the gallery, a buffer task was administered to the participants, which involved filling in a payroll form with their bank details for the payment purposes.

### 2.5. Recognition Test

Following the buffer task an unanticipated object recognition test (similar to the one used in [17,18]) was presented [45] on a laptop with two keys labeled “YES” and “NO”. Subjects were instructed to answer whether they saw the displayed picture in the gallery or not. Accuracy and speed were emphasised in the instruction. Images were shown one at a time in a random sequence, each preceded by a fixation cross on a blank screen lasting for 250 milliseconds. Three additional, unrelated objects were shown at the beginning of the test for the purpose of procedural training. All 14 pictures presented in the gallery were included, with another 14 being new (either completely new or modified versions of the pictures seen in the gallery). For Reaction Time data analysis, only the correct “yes” answers were taken into consideration (78% of the whole dataset). However, the accuracy was also recorded and will be referred to as *RT accuracy*.

Observations lying further than two standard deviations from the mean of the whole dataset were removed [46,47], leaving 69% of all responses valid (*i.e*., correct and within two SD).

The mean value of all valid reaction times calculated for each participant constituted a *Personal Mean RT*. These personal means were later subject to cross-condition comparisons. Mean valueswere also calculated for each picture and for each location, separately contributing to further *picture-oriented* and *location-oriented* analysis. For example, *Mean RT* of picture “N” was equal to 1349 ms (1376 ms in Condition 1 and 1,322 in Condition 2). The same picture’s *Mean RT accuracy* was 0.81 (0.86 in Condition 1 and 0.76 in Condition 2). This means that 81% of all participants correctly recognised picture “N” on the computer screen and reacted by pressing “yes” within two SD of all reaction times in the experimental dataset. The average time of those responses was 1349 ms. This result is irrespective of where the picture was actually seen inside the gallery by each participant and it contributed to *picture-oriented* analysis.

On the contrary, *location-oriented* analysis was conducted by replacing the name of the picture in the dataset by the name of the location at which this picture was seen by each individual participant. Therefore, it was also possible to calculate that, for example, 79% of all participants correctly recognised pictures seen at location “x103” and that they did it after 1411 ms on average. This was irrespective of the actual picture that was present at this location for each individual participant.

### 2.6. Miniature Task (Back-to-the-Wall Measure)

**Figure 6:**
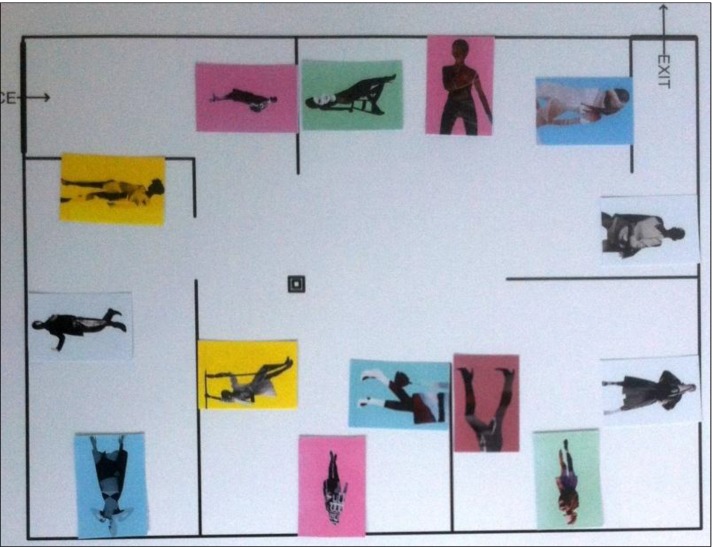
A sample solution of the Miniature Task.

After completing the Recognition Test, participants were asked to move to a table where another task was presented to them, similar to Tour Integration Task [43,48]. They were shown a printed layout of the gallery they had visited and miniature versions of the pictures from the inside. The instruction was to arrange the miniatures on the printed floor plan as they were set out in the gallery. No time limit was suggested. Figure 6 shows a sample solution. For the analysis, the position of each picture miniature was first compared to its true location inside the gallery for each participant. If the participant placed it anywhere along the wall on which the picture was in fact located in the gallery, this participant scored 1 point for this picture. Otherwise 0 was given. Each participant’s score ranging from 0 to 14 was divided by the total number of pictures (14) to create *Personal Mean Back-to-the-Wall* result. Minimum possible value was 0, which would indicate that a participant placed all miniatures on different walls than they were located in the gallery when this person was exploring it. Maximum possible value was 1, which would indicate that this person placed each picture miniature back on the wall he or she saw it on inside. These personal means were later used for cross-condition comparison.

Additionally, mean scores for each picture were calculated equal to the number of correct answers divided by the number of participants. For example, picture “E” (see Figure 1) achieved mean 0.57, indicating that 57% of all participants placed its miniature back on the wall where they saw it in the gallery (54% of participants in Condition 1 and 59% of participants in Condition 2). Furthermore, the data was recoded as a location-oriented variable. That is to say, the name of each picture was changed to the name of the location at which it was positioned for the given participant and all analyses repeated. This allowed for calculation of mean *Back-to-the-Wall* measures for each location.

### 2.7. Salience Study on an Independent Group of Participants

To further control perceived salience of the individual pictures contributing to the gallery experience, a separate online experiment was conducted on an independent group of 54 participants recruited through social network portals. The procedure was designed to imitate the one described by Miller and Carlson [18]. Participants were presented the pool of 14 pictures used in the gallery study and asked to “drag and drop” them on the screen according to “how much they draw your attention”. Because this method would not be feasible for a large number of visual stimuli presented simultaneously on a small computer screen, pictures were displayed in two sets of three and two sets of four. The order of the displayed pictures was fully randomised, and the content (*i.e*., the neighbourhood of other pictures in which each picture appeared) was quasi-randomised in four experimental blocks. Out of 54 participants who took part in the independent salience study, 14 were removed due to not finishing the survey, outlying engagement time, or no “drag and drop” action taken on at least one of four picture sets. The results of the remaining participants (N = 40; 22 female; mean age = 28.74, SD = 8.06; mean time spenton the survey = 189 s, SD = 64 s) were calculated in the following way: for being dragged to the first position within a set, a picture was given the score of 1. For being placed 2nd, 3rd and 4th the scores were 0.66, 0.33 and 0 accordingly for four-picture sets. For the 2nd and 3rd position in a three-picture set 0.5 and 0 points were given. Mean score of each picture was pulled from the positions given to it by each participant and this constituted the *Salience Rating* falling between the range of 0 and 1. In this case a score of 1 would indicate that every participant dragged the picture to the first position along its neighbours. Mean equal to 0 indicated that all participants placed the object at the bottom of the set in which they saw it.

## 3. Results

All statistical analyses were conducted using R [49].

### 3.1. Time Spent Inside

**Figure 7:**
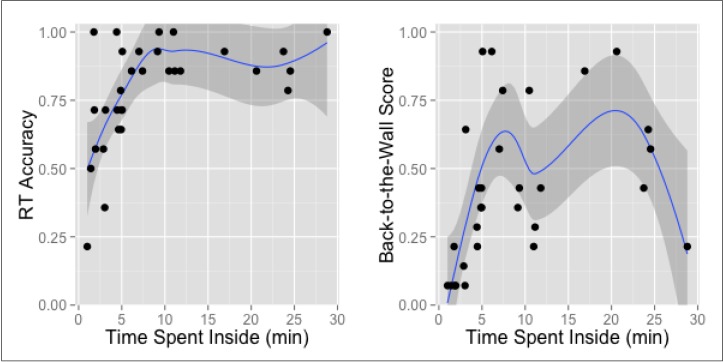
The influence of time spent inside on the personal means of *RT accuracy* and *Back-to-the-Wall score*.

The difference between participants’ *mean time spent inside* the gallery was not significant across the conditions (Condition 1: M = 614 s; SD = 536 s; Condition 2: M = 473 s; SD = 412 s). However, *RT accuracy* increased as the total time spent inside increased for those participants, who spent less than six minutes inside. All participants who stayed inside for longer than 6 minutes correctly recognised between 75% and 100% of pictures. A similar influence of time on shorter visits was observed in the *mean personal Back-to-the-Wall score,* although performance of those staying for longer varied to a greater extent (Figure 7).

### 3.2. Cross-Condition Comparison of Personal Means

Personal Means of *Back-to-the-Wall* score, *RT accuracy, and Mean RT* were subject to cross-condition comparisons.

The difference in *Personal Mean RT* was significant, as indicated by Welch’s *t*-test *t* (25.5) = 2.19; *p* < 0.05 with participants from Condition 1 reacting faster (M = 1,277 ms, S = 296) than those from Condition 2 (M = 1620 ms, SD = 552). Considering that the same pool of pictures was seen in each condition, and that the sequence of this viewing was fully randomised, this result suggests that it was the difference in spatial arrangement that created this effect.

Participants’ *RT accuracy* was similar in both conditions (Condition 1: M = 0.79 ms, SD = 0.19; Condition 2: M = 0.78 ms, SD *=* 0.2) and the difference has not reached statistical significance, similar to other studies using this measure [18,50].

Large variance and flat distribution of *Back-to-the-Wall* results within Condition 2 made it difficult to investigate the cross-conditional difference in Miniature Task results (Condition 1: M = 0.37; σ^2^ = 0.04; Condition 2: M = 0.47; σ^2^ = 0.1; Levene’s test approaching significance at *p* = 0.058). A similar situation occurred in other studies with different tasks measuring spatial knowledge and might be indicative of its very high difficulty in the given context [43], or different strategies acquired by the participants during learning or recall.

### 3.3. Location-Oriented Correlations

Each spatial property derived from picture locations (such as their *isovist size*) can be correlated with location-oriented mean values of the memory tests. Such correlation matrices can be calculated jointly for both conditions (*i.e*., taking into account all locations, from x101 to x214), or for each condition separately, to assess whether the effect holds for both versions of the art gallery. Table 1 presents three correlation matrices divided so. For clarity purposes, correlations between standard Space Syntax measures are omitted.

**Figure 8:**
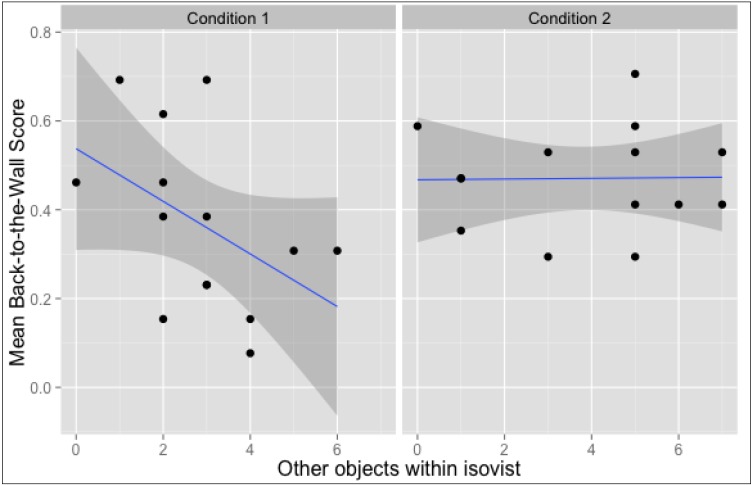
Location-based correlations for *Back-to-the-Wall Score* and the number of other objects within location’s isovist. Data points represent locations (x101-x114 for Condition 1 and x201-x214 for Condition 2).

Please note that the correlation coefficient between *other objects within isovist* and *Back-to-the-Wall* mean scores in Condition 1 was significant when calculated with Spearman’s rank correlation: rho *r_s_*(14) = −0.54; *p* < 0.05, which is less sensible to outliers. This negative correlation means that the more pictures were potentially co-visible with a given location in Condition 1, the less likely participants were to correctly encode spatial information of this location's content. Figure 8 presents the relevant scatter plots.

**Figure 9:**
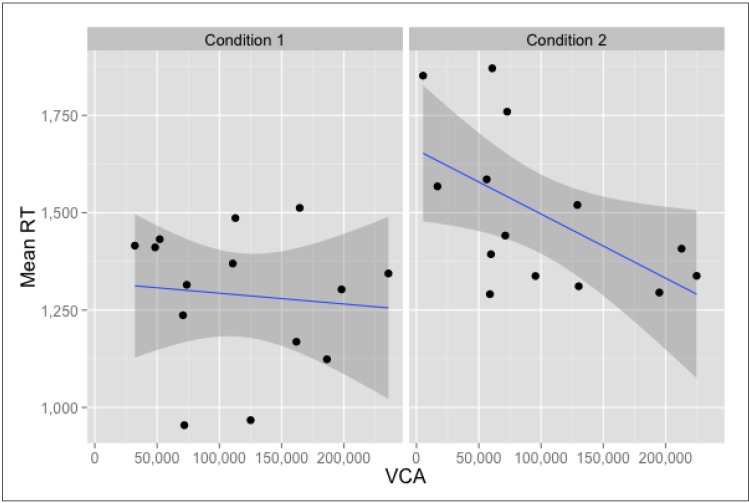
Location-based correlations for *Mean Reaction Time* and *Visibility Catchment Area* (VCA). Data points represent locations.

Another strong negative correlation between *VCA* and *Mean RT* scores occurred in Condition 2, meaning that locations with larger *Visibility Catchment Areas* facilitated faster recognition of pictures seen at those locations. This effect was not present in Condition 1, where *VCAs* were larger on average. Figure 9 presents the relevant scatter plots.

**Table 1:** Correlation matrices for location-oriented variables.

	Both Conditions jointly (locations x101-x214)			Condition 1 (locations x1.)			Condition 2 (locations x2.)		
	*RT*	*RT accuracy*	*Back-to-the-Wall*	*RT*	*RT accuracy*	*Back-to-the-Wall*	*RT*	*RT accuracy*	*Back-to-the-Wall*
RT accuracy	−0.01			0.35			−0.17		
Back-to-the-Wall	0.27	−0.01		0.39	0.06		−0.22	−0.04	
Visibility Catchment Area (VCA)	−0.38 *	−0.12	0.04	−0.1	0.19	−0.12	−0.56*	−0.35	0.41
Other Objects Within Isovist	0.15	−0.11	−0.12	0.11	0.14	−0.47	−0.02	−0.22	0.02
Isovist Area	−0.23	−0.12	−0.1	0.05	0.3	−0.32	−0.42	−0.35	0.18
Connectivity (BVG)	−0.18	−0.03	0.03	0.11	0.46	−0.12	−0.4	−0.31	0.23
Point 2nd Moment	−0.21	−0.09	−0.03	0.1	0.31	−0.14	−0.42	−0.38	0.23

*p* < 0.05 *; *p* < 0.01 **; *p* < 0.001 ***; RT: Reaction Times.

Significant correlations in location-oriented analysis confirm the hypothesis that the effect of space prevails despite the influence of individual artworks’ differences.

### 3.4. Salience Study

Salience Rating for each picture used in the experiment, falling in the range between 0 and 1, was calculated in the independent study, as explained in Section 2.7.

A one-way ANOVA showed that Salience Rating differed significantly between 14 pictures, *F* (13, 546) = 6.44; *p* < 0.001 with picture “D” being rated the most prominent and picture “L” the least. It was then included in the picture-oriented part of the analysis.

### 3.5. Picture-Oriented Analysis

For the picture-oriented analysis, the *Back-to-the-Wall* score correlated with the results of Recognition Test, contrary to the location-oriented analysis. This indicates that *memory of objects* and *memory of their spatial locations* were linked, but it was the object, and not the location, that carried this linkage in participants’ memory.

Also, *Salience Rating* was significantly correlated with mean *Back-to-the-Wall* score of individual pictures (*i.e.*, the ratio of participants who placed the given picture back to the correct wall) in Condition 2. This shows that, in Condition 2, the more visually salient a picture was, the more likely it was that participants placed it back on the correct wall in the Miniature Task following their visit.

Table 2 presents the correlation matrix.

**Table 2:** Correlation matrix for picture-oriented variables.

	Both Conditions jointly			Condition 1			Condition 2		
	*RT*	*RT accuracy*	Back-to-the-Wall	*RT*	*RT accuracy*	Back-to-the-Wall	*RT*	*RT accuracy*	*Back-to-the-Wall*
RT accuracy	−0.57 *			−0.17			−0.52		
Back-to-the-Wall	−0.64 *	0.78 ***		−0.25	0.73 **		−0.39	0.32	
Salience Rating	−0.26	0.2	0.32	−0.17	0.24	0.01	−0.21	0.14	0.56 *

*p* < 0.05 *; *p* < 0.01 **; *p* < 0.001 ***; RT: Reaction Times.

## 4. Discussion

Time spent inside significantly correlated with both types of participants’ memory (*i.e*., their spatial memory indicated by *Back-to-the-Wall* scores, and their memory for objects, shown in *RT accuracy*). The factor of time seems to only be beneficial to participants who spent less than nine minutes inside the gallery. This suggests that for this particular gallery space, staying inside for longer than that did not help to further increase visitors’ spontaneous memory. This might be the result of fatigue, reaching the limit of memory capacity, or lost interest for no-longer-novel stimuli. In each case, this finding would be strictly case-specific and the time’s threshold value should differ for other museum types, bigger or smaller surface areas, different artworks being displayed, or higher/lower number of stimuli present in space. Importantly, this effect is probably moderated by task instruction: despite the study’s instruction to “explore as you would explore a regular art gallery,” the awareness of taking part in a psychological study may have modified the peace of participants’ spontaneous memorisation. Caution should be given when relating this result to any other art gallery situation, yet the mere existence of such a threshold value seems worthy of future investigations.

The correlation between the results of both memory tests (the computer recognition test and the Back-to-the-Wall score) in the *picture-oriented* analysis was not mirrored in the *location-oriented* calculations. This can be interpreted in the following way: the pictures which were recognised faster and more accurately on the computer recognition test tended to be placed back on the correct wall more often in the Miniature Task. If, however, participants could correctly (and quickly) recognise pictures from, say, location x108, it did not mean that content of this location would automatically be easier to recall in the Miniature Task. Hence, it seems that in this study, the objects, and not locations, were the carriers of linked object-based and space-based information, although this result might be context-specific [15]. Once a picture was well remembered, so was the spatial information relevant to it (although not necessarily in this chronological order). The role of spatial configuration in this situation is to facilitate the uptake of this information by exposing pictures in a particular way. Even the most prominent locations, however, would not guarantee that both types of memory traces would be enhanced for pictures placed on them. This again signifies the importance of separating both types of memory for objects in real-life spaces. Further analyses would shed more light on this interrelation.

Condition 1 resulted in faster *Reaction Times* than viewing the same artworks in Condition 2. Location-oriented reaction times were significantly correlated with the size of the *Visibility Catchment Area*. This effect was, however, only valid for Condition 2, whose participants performed worse on the task and whereby the average *VCA* was smaller. Smaller mean *VCA* is the consequence of many pictures being located close to room corners, or with a restricted space in front of them. A possible explanation of the result is that, assuming any random walking path through the environment, higher mean *VCA* indicates that the pictures had higher probability of falling within a comfortable viewing zone of each visitor for longer. This seems not to play a significant role when viewing conditions are comfortable (large *VCA*, separated pictures in Condition 1), but only to become an inhibiting factor when *VCAs* become severely limited. The relation therefore seems to be non-linear, and there might be a certain threshold of average-VCA-to-area ratio involved, which constitutes the boundary between comfortable and uncomfortable viewing conditions.

It is important to note that other spatial factors not investigated in current paper might be involved in this effect. *VCA* was analysed in detail because it is descriptive of many other potentially disrupting spatial properties while expressing the quality of the viewing experience regardless of other artworks present in space. This qualitatively distinguishes it from measures of potential co-visibility (such as *other objects within isovist*) which are, by definition, indicative of the relations between many objects in space. In Condition 1, the results of the Miniature Task were negatively correlated with the *number of objects within the location’s isovist*. The number of other objects within the isovist is indicative of the possible co-visibility of other objects during the investigation of a single picture. Since the *Back-to-the-Wall* measure was designed to establish the memory performance for inter-object relations, it seems counterintuitive that noticing other objects around would inhibit successful completion of the task. Perhaps a higher likelihood of being distracted is not beneficial for establishing links between objects and their locations in space. Such an explanation could also clarify the large spread of this variable’s results among participants from Condition 2.

An independently assessed Salience Rating shed more light on this effect. The significant correlation of the *Salience Rating* with *Back-to-the-Wall* score in Condition 2 indicates that it was the picture’s salience that drove participants’ spontaneous spatial memory in the situation in which space was designed to interrupt comfortable viewing rather than help it. A suggested explanation is that Condition 1 allowed for an easy one-to-one mapping between the object and its spatial unit, which caused faster reaction times in the Recognition Test. This would remain in line with Janzen’s [17] interpretation of her own results. When spatial relations become less obvious, perhaps confusing, and when the potential for distraction rises, perceived salience starts to play a significant role in directing human spontaneous memory in a gallery setting. This proves that in the described context, the effect of spatial arrangement on human memory is significant, although nonlinear.

Whether there is any qualitative difference between art gallery set-ups designed according to two very different spatial strategies is unclear. However, as different patterns of location-based correlations across the conditions show, the impact of spatial layout does not operate on a location-by-location basis, but only in conjunction with nearby objects. This might be happening on the level of vista-spaces [51], and the whole environment. The probability of encountering more complex vistas varied across the conditions, perhaps accumulating to the level at which it is almost impossible to encounter non-complex vistas in Condition 2. Such a situation could constitute a “threshold” between the visual quality of two spatial set-ups.

## 5. Conclusions

It is important to emphasise that a comfortable (or “cognitively efficient”) space itself does not seem to greatly help in enhancing the memory, but a badly designed one can become a major inhibiting factor. However, the current data does not determine where the boundaries of “badly designed gallery” lie. It seems evident, however, that such a badly designed space can shift the potential outcome of a cautiously prepared, curated exhibition into one driven mainly by the objects’ salience. At the same time, however, it need not be ignored that creating such a situation might form part of an artist’s intention [52]. Therefore, it would be difficult to suggest design practices leading to the emergence of a *perfect* exhibition space. It is much more realistic, and potentially useful, to propose how to consciously control such spaces and how to avoid their unwanted configuration. Spatial analyses presented in this work can contribute to the curator’s understanding of the potential cognitive impact of their exhibitions.
